# Supplementary material for: Factors associated with mechanical device-related complications in tube fed patients: A multicenter prospective cohort study
Source: PLoS One. 2020 Nov 19;15(11):e0241849. doi: 10.1371/journal.pone.0241849 (PMC7676660; doi:10.1371/journal.pone.0241849)
Supplement: S1 File — (PDF) [file pone.0241849.s002.pdf]

I - FORMULÁRIO DE COLETA DE DADOS GERAL

A - DADOS GERAIS

1. Número do paciente da pesquisa:

2. 2. (DC_Ad)Data da Coleta:

Data

Data

DD/MM/AAAA

3. Hospital:

- HCA
- HGF
- HCFMRP-USP
- HEAB
- HES
- HSVP
- HSCRGS

4. Especialidade:

- Cardiologia
- Clínica Geral
- Dermatologia
- Endocrinologia
- Gastroenterologia
- Geriatria
- Hematologia
- Imunologia
- Moléstias infectocontagiosas
- Nefrologia
- Neurologia
- Oncologia
- Pneumologia
- Unidade Metabólica
- Outro (especifique)

5. Data de admissão na Unidade/Enfermaria:

Data

Data

DD/MM/AAAA

6. Período da admissão do paciente na Unidade/Enfermaria:

- Manhã
- Tarde
- Noite

7. Em uso de sonda enteral na admissão na Unidade/Enfermaria?

- Sim
- Não

I - FORMULÁRIO DE COLETA DE DADOS GERAL

B – DADOS SOCIOECONÔMICOS E DEMOGRÁFICOS

8. Número do registro:

9. Data de nascimento:

Data

Data

DD/MM/AAAA

10. Sexo:

- Feminino
- Masculino
- NR/NS

11. Procedência:

12. Estado:

- ◯ Acre
- ◯ Alagoas
- ◯ Amapá
- ◯ Amazonas
- ◯ Bahia
- ◯ Ceará
- ◯ Distrito Federal
- ◯ Espírito Santo
- ◯ Goiás
- ◯ Maranhão
- ◯ Mato Grosso
- ◯ Mato Grosso do Sul
- ◯ Minas Gerais
- ◯ Pará
- ◯ Paraíba
- ◯ Paraná
- ◯ Pernambuco
- ◯ Piauí
- ◯ Rio de Janeiro
- ◯ Rio Grande do Norte
- ◯ Rio Grande do Sul
- ◯ Rondônia
- ◯ Roraima
- ◯ Santa Catarina
- ◯ São Paulo
- ◯ Sergipe
- ◯ Tocantins
- ◯ Não se aplica

13. País

- ◯ Brasil
- ◯ Outro (especifique)

14. Em relação a sua cor/raça, o sr(a) se considera...

- Branca
- Negra
- Parda
- Indígena
- Amarela (oriental)
- NR/NS

15. Qual o estado civil atual?

- Casado
- Solteiro
- Separado/divorciado
- Viúvo
- Companheiro
- Outro (especifique)

16. O(A) Sr(a) estudou na escola?

- Não
- Não, mas sabe ler e escrever
- Ensino fundamental incompleto
- Ensino fundamental completo
- Ensino médio incompleto
- Ensino médio completo
- Nível superior incompleto
- Nível superior completo
- Pós-graduação
- Pós-graduação incompleta

17. Qual a sua ocupação atual?

C – DADOS CLÍNICOS E FISIOLÓGICOS NA ADMISSÃO

18. (CIDP_Ad)CID-10 Principal:

19. (CIDS_Ad)Comorbidade(s) ou CID secundário(s):

- [ ] Infarto do miocárdio
- [ ] Insuficiência cardíaca congestiva
- [ ] Doença vascular periférica / Hipertensão arterial
- [ ] Doença cérebro-vascular
- [ ] Demência
- [ ] Doença pulmonar crônica
- [ ] Doença do tecido conjuntivo
- [ ] Úlcera
- [ ] Doença crônica do fígado e cirrose
- [ ] Diabetes sem complicação
- [ ] Hemiplegia ou paraplegia
- [ ] Doença renal severa ou moderada
- [ ] Diabetes com complicação
- [ ] Tumor
- [ ] Leucemia
- [ ] Linfoma
- [ ] Doença do fígado severa ou moderada
- [ ] Tumor maligno ou metástase
- [ ] AIDS
- [ ] Outro (especifique)

20. (Fug_Ad) Resultado Fugulin:

- Cuidado mínimo
- Cuidado intermediário
- Cuidado alta-dependência
- Cuidado semi-intensivo
- Cuidado intensivo
- Não se aplica

21. Exames Laboratoriais na Admissão:

(Creat_Ad) Creatinina (Cr)

(Ureia_Ad) Ureia (Ur)

(TGO_Ad) Transaminase Glutâmico Oxalacético (TGO)

(TGP_Ad) Transaminase Glutâmico Pirúvica (TGP; SGPT)

(Fosfa_Ad) Fosfatase Alcalina

22. Dados Fisiológicos na Admissão:

(FC_Ad) Frequência Cardíaca (bpm)

(FR_Ad) Frequência Respiratória (ipm)

(PAS_Ad) Pressão Arterial Sistólica (mmHg)

(Temp_Ad) Temperatura (°C)

(Sat_Ad) Saturação de O2 (%)

23. (Ncons_Ad)Nível de consciência:

- Alerta
- Confuso
- Resposta à dor
- Inconsciente

24. (Rdis_Ad)Respiração com dispositivo invasivo (TOT, traqueostomia)?

- Sim
- Não

D – DADOS TERAPÊUTICOS

Dados relacionados à sonda enteral:

25. *D*ata do início do uso da sonda enteral na enfermaria, pela primeira vez:

Data

Data

DD/MM/AAAA

26. Motivo para o uso da sonda enteral:

- Disfagia
- Uso de tubo orotraqueal
- Queda do sensório / rebaixamento do nível de consciência / queda do estado geral
- Desnutrição
- Inapetência / baixa aceitação via oral
- Outro (especifique)

27. Material da sonda:

- PVC (Levine)
- Poliuretano
- Silicone
- Outro (especifique)

28. Calibre da sonda:

- 8 fr
- 10 fr
- 12 fr
- 14 fr
- 16 fr
- 18 fr
- 20 fr
- 22 fr
- Outro (especifique)

29. Fabricante da sonda:

- Embramed®
- Brasmed®
- Covidien®
- Freka®
- Frenesius®
- Markmed®
- Medsonda®
- Medicone®
- Silmag®
- Solumed®
- Tylor®
- Outro (especifique)

30. A sonda de poliuretano é radiopaca?

- Sim
- Não
- Não se aplica

31. A sonda foi introduzida por qual cavidade?

- Oral
- Nasal

32. Técnica utilizada para introdução da sonda:

- ◯ Às cegas / beira leito
- ◯ Endoscopia
- ◯ Fluoroscopia
- ◯ Ultrassom
- ◯ Não se aplica
- ◯ Outro (especifique)

33. Posição da extremidade distal da sonda:

- ◯ Gástrica
- ◯ Entérica (duodenal/jejunal)
- ◯ Não se aplica

34. Confirmado posicionamento da sonda após a introdução?

- ◯ Sim
- ◯ Não
- ◯ Não se aplica

35. Técnica(s) utilizada(s) para confirmar posicionamento da sonda, após a introdução:

- ◯ Somente ausculta epigástrica
- ◯ Ausculta epigástrica e aspiração do conteúdo gástrico
- ◯ Somente aspiração do conteúdo gástrico
- ◯ Somente exame radiológico (raio X)
- ◯ Ausculta epigástrica e exame radiológico (raio X)
- ◯ Ausculta epigástrica, aspiração do conteúdo gástrico e exame radiológico (raio X)
- ◯ Não se aplica
- ◯ Outro (especifique)

36. Resultado do laudo do Raio X de confirmação do posicionamento da extremidade distal da sonda:

- ◯ Gástrica
- ◯ Entérica
- ◯ Esôfago
- ◯ Traqueia
- ◯ Pulmão
- ◯ Mediastino
- ◯ Espaço pleural
- ◯ Não realizado
- ◯ Não se aplica
- ◯ Outro (especifique)

37. Prescrição de dieta por via oral?

- ◯ Sim
- ◯ Não

D – DADOS TERAPÊUTICOS

Dados relacionados à prescrição da dieta na admissão:

38. (Tdiet_Ad)Tipo de dieta:

- ○ Padrão
- ○ Polimérica
- ○ Oligomérica
- ○ Hipercalórica
- ○ Com fibras
- ○ Especializada
- ○ Monomérica ou elementar
- ○ Modular
- ○ Não se aplica
- ○ Outro (especifique)

39. (Metad_Ad)Método de administração da dieta enteral:

- ○ Sistema aberto ou intermitente
- ○ Sistema fechado ou contínuo
- ○ Não se aplica

40. (Tinf_Ad)Tipo de infusão da dieta enteral:

- ○ Gravitacional (gotejamento)
- ○ Bomba de infusão
- ○ Não se aplica
- ○ Outro (especifique)

41. (Pnot_Ad)Há pausa noturna da dieta enteral?

- ○ Sim
- ○ Não
- ○ Não se aplica

42. (Vt24h_Ad)Volume total da dieta enteral em 24 horas (ml):

43. (Cal24h_Ad)Calorias totais da dieta enteral em 24 horas (Cal):

44. (Fadm_Ad)Frequência de administração:

D – DADOS TERAPÊUTICOS

Dados relacionados aos medicamentos prescritos na admissão:

45. Medicamento 1

Nome

Apresentação

Dose

Aprazamento

46. Dados do Medicamento 1

	Forma Farmacêutica	Via de administração	Frequência
Medicamento 1			

47. Medicamento 2

Nome

Apresentação

Dose

Aprazamento

48. Dados do Medicamento 2

	Forma Farmacêutica	Via de administração	Frequência
Medicamento 2			

49. Medicamento 3

Nome

Apresentação

Dose

Aprazamento

50. Dados do Medicamento 3

	Forma Farmacêutica	Via de administração	Frequência
Medicamento 3			

51. Medicamento 4

Nome

Apresentação

Dose

Aprazamento

52. Dados do Medicamento 4

	Forma Farmacêutica	Via de administração	Frequência
Medicamento 4			

53. Medicamento 5

Nome

Apresentação

Dose

Aprazamento

54. Dados do Medicamento 5

	Forma Farmacêutica	Via de administração	Frequência
Medicamento 5			

55. Medicamento 6

Nome

Apresentação

Dose

Aprazamento

56. Dados do Medicamento 6

	Forma Farmacêutica	Via de administração	Frequência
Medicamento 6			

57. Medicamento 7

Nome

Apresentação

Dose

Aprazamento

58. Dados do Medicamento 7

	Forma Farmacêutica	Via de administração	Frequência
Medicamento 7			

59. Medicamento 8

Nome

Apresentação

Dose

Aprazamento

60. Dados do Medicamento 8

	Forma Farmacêutica	Via de administração	Frequência
Medicamento 8			

61. Medicamento 9

Nome

Apresentação

Dose

Aprazamento

62. Dados do Medicamento 9

	Forma Farmacêutica	Via de administração	Frequência
Medicamento 9			

63. Medicamento 10

Nome

Apresentação

Dose

Aprazamento

64. Dados do Medicamento 10

	Forma Farmacêutica	Via de administração	Frequência
Medicamento 10			

65. Medicamento 11

Nome

Apresentação

Dose

Aprazamento

66. Dados do Medicamento 11

	Forma Farmacêutica	Via de administração	Frequência
Medicamento 11			

67. Medicamento 12

Nome

Apresentação

Dose

Aprazamento

68. Dados do Medicamento 12

	Forma Farmacêutica	Via de administração	Frequência
Medicamento 12			

69. Medicamento 13

Nome

Apresentação

Dose

Aprazamento

70. Dados do Medicamento 13

	Forma Farmacêutica	Via de administração	Frequência
Medicamento 13			

71. Medicamento 14

Nome

Apresentação

Dose

Aprazamento

72. Dados do Medicamento 14

	Forma Farmacêutica	Via de administração	Frequência
Medicamento 14			

73. Medicamento 15

Nome

Apresentação

Dose

Aprazamento

74. Dados do Medicamento 15

	Forma Farmacêutica	Via de administração	Frequência
Medicamento 15			

75. Medicamento 16

Nome

Apresentação

Dose

Aprazamento

76. Dados do Medicamento 16

	Forma Farmacêutica	Via de administração	Frequência
Medicamento 16			

77. Medicamento 17

Nome

Apresentação

Dose

Aprazamento

78. Dados do Medicamento 17

	Forma Farmacêutica	Via de administração	Frequência
Medicamento 17			

79. Medicamento 18

Nome

Apresentação

Dose

Aprazamento

80. Dados do Medicamento 18

	Forma Farmacêutica	Via de administração	Frequência
Medicamento 18			

81. Medicamento 19

Nome

Apresentação

Dose

Aprazamento

82. Dados do Medicamento 19

	Forma Farmacêutica	Via de administração	Frequência
Medicamento 19			

83. Medicamento 20

Nome

Apresentação

Dose

Aprazamento

84. Dados do Medicamento 20

	Forma Farmacêutica	Via de administração	Frequência
Medicamento 20			

85. Medicamento 21

Nome

Apresentação

Dose

Aprazamento

86. Dados do Medicamento 21

	Forma Farmacêutica	Via de administração	Frequência
Medicamento 21			

87. Medicamento 22

Nome

Apresentação

Dose

Aprazamento

88. Dados do Medicamento 22

	Forma Farmacêutica	Via de administração	Frequência
Medicamento 22			

89. Medicamento 23

Nome

Apresentação

Dose

Aprazamento

90. Dados do Medicamento 23

	Forma Farmacêutica	Via de administração	Frequência
Medicamento 23			

91. Medicamento 24

Nome

Apresentação

Dose

Aprazamento

92. Dados do Medicamento 24

	Forma Farmacêutica	Via de administração	Frequência
Medicamento 24			

93. Medicamento 25

Nome	
Apresentação	
Dose	
Aprazamento	

94. Dados do Medicamento 25

	Forma Farmacêutica	Via de administração	Frequência
Medicamento 25			

I - FORMULÁRIO DE COLETA DE DADOS GERAL

95. Observação:

