# Supplementary material for: Factors associated with mechanical device-related complications in tube fed patients: A multicenter prospective cohort study
Source: PLoS One. 2020 Nov 19;15(11):e0241849. doi: 10.1371/journal.pone.0241849 (PMC7676660; doi:10.1371/journal.pone.0241849)
Supplement: S2 File — (PDF) [file pone.0241849.s003.pdf]

II - FORMULÁRIO INCIDENTES RELACIONADOS À SONDA ENTERAL

A - DADOS GERAIS

1. Número do paciente na pesquisa:

2. Número do registro:

3. Data e horário do Incidente:

(DC_Inc)Data

Data | Hora | AM/PM

DD/MM/AAAA | hh | mm | -

II - FORMULÁRIO INCIDENTES RELACIONADOS À SONDA ENTERAL

B – DADOS CLÍNICOS E FISIOLÓGICOS NO MOMENTO DO INCIDENTE

4. (CIDP_iS) CID-10 Principal:

5. (CIDS_iS) Comorbidade(s) ou CID secundário(s):

- [ ] Infarto do miocárdio
- [ ] Insuficiência cardíaca congestiva
- [ ] Doença vascular periférica / Hipertensão arterial
- [ ] Doença cérebro-vascular
- [ ] Demência
- [ ] Doença pulmonar crônica
- [ ] Doença do tecido conjuntivo
- [ ] Úlcera
- [ ] Doença crônica do fígado e cirrose
- [ ] Diabetes sem complicação
- [ ] Hemiplegia ou paraplegia
- [ ] Doença renal severa ou moderada
- [ ] Diabetes com complicação
- [ ] Tumor
- [ ] Leucemia
- [ ] Linfoma
- [ ] Doença do fígado severa ou moderada
- [ ] Tumor maligno ou metástase
- [ ] AIDS
- [ ] Outro (especifique)

6. (Fug_iS) Resultado Fugulin:

- ○ Cuidado mínimo
- ○ Cuidado intermediário
- ○ Cuidado alta-dependência
- ○ Cuidado semi-intensivo
- ○ Cuidado intensivo

7. **Dados Fisiológicos:**

(FC_iS) Frequência Cardíaca (bpm) ____________________

(FR_iS) Frequência Respiratória (ipm) ____________________

(PAS_iS) Pressão Arterial Sistólica (mmHg) ____________________

(Temp_iS) Temperatura (°C) ____________________

(Sat_iS) Saturação de O2 (%) ____________________

8. (Ncons_iS) Nível de consciência:

- ○ Alerta
- ○ Confuso
- ○ Resposta à dor
- ○ Inconsciente

9. (Rdis_iS) Respiração com dispositivo invasivo (TOT, traqueostomia)?

- ○ Sim
- ○ Não

10. Com acompanhante no momento do incidente?

- ○ Sim
- ○ Não
- ○ NR/NS

II - FORMULÁRIO INCIDENTES RELACIONADOS À SONDA ENTERAL

C – DADOS TERAPÊUTICOS NO MOMENTO DO INCIDENTE

Dados relacionados à prescrição da dieta enteral:

11. (Tdiet_iS)Tipo de dieta:

- ◯ Padrão
- ◯ Polimérica
- ◯ Oligomérica
- ◯ Hipercalórica
- ◯ Com fibras
- ◯ Especializada
- ◯ Monomérica ou elementar
- ◯ Modular
- ◯ Não se aplica
- ◯ Outro (especifique)

[ ]

12. (Metad_iS)Método de administração da dieta:

- ◯ Sistema aberto ou intermitente
- ◯ Sistema fechado ou contínuo
- ◯ Não se aplica

13. (Tinf_iS)Tipo de infusão da dieta:

- ◯ Gravitacional (gotejamento)
- ◯ Bomba de infusão
- ◯ Não se aplica
- ◯ Outro (especifique)

[ ]

14. (Pnot_iS)Há pausa noturna da dieta enteral?

- ◯ Sim
- ◯ Não
- ◯ Não se aplica

15. (Vt24h_iS)Volume total da dieta enteral em 24 horas (ml):

[ ]

16. (Cal24h_iS)Calorias totais da dieta enteral em 24 horas (Cal):

17. (Fadm_iS)Frequência de administração:

II - FORMULÁRIO INCIDENTES RELACIONADOS À SONDA ENTERAL

C – DADOS TERAPÊUTICOS NO MOMENTO DO INCIDENTE

Dados relacionados aos medicamentos prescritos:

18. (Inc)Medicamento 1

Nome

Apresentação

Dose

Aprazamento

19. (Inc)Dados Medicamento 1

	(Inc)Forma farmacêutica	(Inc)Via de administração	(Inc)Frequência
(Inc)Medicamento 1			

20. (Inc)Medicamento 2

Nome

Apresentação

Dose

Aprazamento

21. (Inc)Dados Medicamento 2

	(Inc)Forma farmacêutica	(Inc)Via de administração	(Inc)Frequência
(Inc)Medicamento 2			

22. (Inc)Medicamento 3

Nome

Apresentação

Dose

Aprazamento

23. (Inc)Dados Medicamento 3

	(Inc)Forma farmacêutica	(Inc)Via de administração	(Inc)Frequência
(Inc)Medicamento 3			

24. (Inc)Medicamento 4

Nome

Apresentação

Dose

Aprazamento

25. (Inc)Dados Medicamento 4

	(Inc)Forma farmacêutica	(Inc)Via de administração	(Inc)Frequência
(Inc)Medicamento 4			

26. (Inc)Medicamento 5

Nome

Apresentação

Dose

Aprazamento

27. (Inc)Dados Medicamento 5

	(Inc)Forma farmacêutica	(Inc)Via de administração	(Inc)Frequência
(Inc)Medicamento 5			

28. (Inc)Medicamento 6

Nome

Apresentação

Dose

Aprazamento

29. (Inc)Dados Medicamento 6

	(Inc)Forma farmacêutica	(Inc)Via de administração	(Inc)Frequência
(Inc)Medicamento 6			

30. (Inc)Medicamento 7

Nome

Apresentação

Dose

Aprazamento

31. (Inc)Dados Medicamento 7

	(Inc)Forma farmacêutica	(Inc)Via de administração	(Inc)Frequência
(Inc)Medicamento 7			

32. (Inc)Medicamento 8

Nome

Apresentação

Dose

Aprazamento

33. (Inc)Dados Medicamento 8

	(Inc)Forma farmacêutica	(Inc)Via de administração	(Inc)Frequência
(Inc)Medicamento 8			

34. (Inc)Medicamento 9

Nome

Apresentação

Dose

Aprazamento

35. (Inc)Dados Medicamento 9

	(Inc)Forma farmacêutica	(Inc)Via de administração	(Inc)Frequência
(Inc)Medicamento 9			

36. (Inc)Medicamento 10

Nome

Apresentação

Dose

Aprazamento

37. (Inc)Dados Medicamento 10

	(Inc)Forma farmacêutica	(Inc)Via de administração	(Inc)Frequência
(Inc)Medicamento 10			

38. (Inc)Medicamento 11

Nome

Apresentação

Dose

Aprazamento

39. (Inc)Dados Medicamento 11

	(Inc)Forma farmacêutica	(Inc)Via de administração	(Inc)Frequência
(Inc)Medicamento 11			

40. (Inc)Medicamento 12

Nome

Apresentação

Dose

Aprazamento

41. (Inc)Dados Medicamento 12

	(Inc)Forma farmacêutica	(Inc)Via de administração	(Inc)Frequência
(Inc)Medicamento 12			

42. (Inc)Medicamento 13

Nome

Apresentação

Dose

Aprazamento

43. (Inc)Dados Medicamento 13

	(Inc)Forma farmacêutica	(Inc)Via de administração	(Inc)Frequência
(Inc)Medicamento 13			

44. (Inc)Medicamento 14

Nome

Apresentação

Dose

Aprazamento

45. (Inc)Dados Medicamento 14

	(Inc)Forma farmacêutica	(Inc)Via de administração	(Inc)Frequência
(Inc)Medicamento 14			

46. (Inc)Medicamento 15

Nome	
Apresentação	
Dose	
Aprazamento	

47. (Inc)Dados Medicamento 15

	(Inc)Forma farmacêutica	(Inc)Via de administração	(Inc)Frequência
(Inc)Medicamento 15			

48. (Inc)Medicamento 16

Nome	
Apresentação	
Dose	
Aprazamento	

49. (Inc)Dados Medicamento 16

	(Inc)Forma farmacêutica	(Inc)Via de administração	(Inc)Frequência
(Inc)Medicamento 16			

50. (Inc)Medicamento 17

Nome	
Apresentação	
Dose	
Aprazamento	

51. (Inc)Dados Medicamento 17

	(Inc)Forma farmacêutica	(Inc)Via de administração	(Inc)Frequência
(Inc)Medicamento 17			

52. (Inc)Medicamento 18

Nome

Apresentação

Dose

Aprazamento

53. (Inc)Dados Medicamento 18

	(Inc)Forma farmacêutica	(Inc)Via de administração	(Inc)Frequência
(Inc)Medicamento 18			

54. (Inc)Medicamento 19

Nome

Apresentação

Dose

Aprazamento

55. (Inc)Dados Medicamento 19

	(Inc)Forma farmacêutica	(Inc)Via de administração	(Inc)Frequência
(Inc)Medicamento 19			

56. (Inc)Medicamento 20

Nome

Apresentação

Dose

Aprazamento

57. (Inc)Dados Medicamento 20

	(Inc)Forma farmacêutica	(Inc)Via de administração	(Inc)Frequência
(Inc)Medicamento 20			

58. (Inc)Medicamento 21

Nome

Apresentação

Dose

Aprazamento

59. (Inc)Dados Medicamento 21

	(Inc)Forma farmacêutica	(Inc)Via de administração	(Inc)Frequência
(Inc)Medicamento 21			

60. (Inc)Medicamento 22

Nome

Apresentação

Dose

Aprazamento

61. (Inc)Dados Medicamento 22

	(Inc)Forma farmacêutica	(Inc)Via de administração	(Inc)Frequência
(Inc)Medicamento 22			

62. (Inc)Medicamento 23

Nome

Apresentação

Dose

Aprazamento

63. (Inc)Dados Medicamento 23

	(Inc)Forma farmacêutica	(Inc)Via de administração	(Inc)Frequência
(Inc)Medicamento 23			

64. (Inc)Medicamento 24

Nome

Apresentação

Dose

Aprazamento

65. (Inc)Dados Medicamento 24

	(Inc)Forma farmacêutica	(Inc)Via de administração	(Inc)Frequência
(Inc)Medicamento 24			

66. (Inc)Medicamento 25

Nome

Apresentação

Dose

Aprazamento

67. (Inc)Dados Medicamento 25

	(Inc)Forma farmacêutica	(Inc)Via de administração	(Inc)Frequência
(Inc)Medicamento 25			

II - FORMULÁRIO INCIDENTES RELACIONADOS À SONDA ENTERAL

D – INCIDENTE(S) RELACIONADO(S) À SONDA ENTERAL

68. Incidente mecânico:

- ◯ Saque não planejado / acidental
- ◯ Obstrução
- ◯ Migração / deslocamento
- ◯ Epistaxe / sangramento nasal
- ◯ Edema de mucosa nasal
- ◯ Perfuração ou estenose de esôfago
- ◯ Perfuração de cérebro
- ◯ Pneumotórax
- ◯ Várias tentativas de introdução da sonda
- ◯ Não se aplica

II - FORMULÁRIO INCIDENTES RELACIONADOS À SONDA ENTERAL

D – INCIDENTE(S) RELACIONADO(S) À SONDA ENTERAL

69. Incidente Metabólico:

- ◯ Hipernatremia
- ◯ Hiponatremia
- ◯ Hiperglicemia
- ◯ Hipoglicemia
- ◯ Não se aplica

II - FORMULÁRIO INCIDENTES RELACIONADOS À SONDA ENTERAL

D – INCIDENTE(S) RELACIONADO(S) À SONDA ENTERAL

70. Incidente gastrointestinal:

- ◯ Náusea / vômito
- ◯ Diarreia
- ◯ Constipação / Obstipação
- ◯ Cólica / Distensão abdominal / Flatulência
- ◯ Não se aplica

II - FORMULÁRIO INCIDENTES RELACIONADOS À SONDA ENTERAL

D – INCIDENTE(S) RELACIONADO(S) À SONDA ENTERAL

71. Incidente infeccioso:

- ○ Pneumonia aspirativa
- ○ Gastroenterocolite
- ○ Não se aplica

D – INCIDENTE(S) RELACIONADO(S) À SONDA ENTERAL

72. Outro incidente relacionado a sonda enteral:

- ○ Lesão da pele associada à fixação
- ○ Broncoaspiração
- ○ Conexão errada
- ○ Qualidade do material da sonda
- ○ Não se aplica
- ○ Outro (especifique)

II - FORMULÁRIO INCIDENTES RELACIONADOS À SONDA ENTERAL

D – INCIDENTE(S) RELACIONADO(S) À SONDA ENTERAL

73. Descrever, em ordem cronológica (incluindo fatos e horários), como ocorreu o incidente:

74. Consequência do incidente para paciente:

- ◯ Nenhum
- ◯ Leve
- ◯ Moderado
- ◯ Grave
- ◯ Óbito

75. Medida(s) adotada(s) após o incidente:

- ☐ Comunicado ao enfermeiro/ equipe de enfermagem
- ☐ Comunicado ao médico
- ☐ Comunicado à família / cuidador
- ☐ Exame físico
- ☐ Registrado no prontuário
- ☐ Aferidos sinais vitais
- ☐ Repassada sonda / passada nova sonda
- ☐ Comunicado ao Núcleo de Segurança do Paciente
- ☐ Nenhuma
- ☐ Outro (especifique)

76. Fonte(s) de informação sobre a ocorrência do incidente:

- ☐ Equipe de enfermagem
- ☐ Equipe médica
- ☐ Paciente
- ☐ Cuidador / Acompanhante
- ☐ Outro (especifique)

77. Meio(s) de obtenção da informação sobre a ocorrência do incidente:

- [ ] Caderneta
- [ ] Prontuário
- [ ] Relato verbal
- [ ] Sistema de notificação
- [ ] Outro (especifique)

II - FORMULÁRIO INCIDENTES RELACIONADOS À SONDA ENTERAL

78. Observação:
