# Supplementary material for: Factors associated with mechanical device-related complications in tube fed patients: A multicenter prospective cohort study
Source: PLoS One. 2020 Nov 19;15(11):e0241849. doi: 10.1371/journal.pone.0241849 (PMC7676660; doi:10.1371/journal.pone.0241849)
Supplement: S3 File — (PDF) [file pone.0241849.s004.pdf]

III - INÍCIO DO USO DA SONDA E ACOMPANHAMENTO DO PACIENTE

A - DADOS GERAIS

1. Número do paciente na pesquisa:

2. Número do registro:

III - INÍCIO DO USO DA SONDA E ACOMPANHAMENTO DO PACIENTE

B - VARIÁVEIS CLÍNICAS

Início do uso da sonda:

3. (DiuS)Data:

Data

Data

DD/MM/AAAA

4. (CIDP_Ac_iniS) CID-10 Principal:

5. (CIDS_Ac_iniS) Comorbidade(s) ou CID secundário(s):

- [ ] Infarto do miocárdio
- [ ] Insuficiência cardíaca congestiva
- [ ] Doença vascular periférica / Hipertensão arterial
- [ ] Doença cérebro-vascular
- [ ] Demência
- [ ] Doença pulmonar crônica
- [ ] Doença do tecido conjuntivo
- [ ] Úlcera
- [ ] Doença crônica do fígado e cirrose
- [ ] Diabetes sem complicação
- [ ] Hemiplegia ou paraplegia
- [ ] Doença renal severa ou moderada
- [ ] Diabetes com complicação
- [ ] Tumor
- [ ] Leucemia
- [ ] Linfoma
- [ ] Doença do fígado severa ou moderada
- [ ] Tumor maligno ou metástase
- [ ] AIDS
- [ ] Outro (especifique)

6. (Fug_Ac_iniS) Resultado Fugulin:

- ◯ Cuidado mínimo
- ◯ Cuidado intermediário
- ◯ Cuidado alta-dependência
- ◯ Cuidado semi-intensivo
- ◯ Cuidado intensivo

7. Exames Laboratoriais:

(Creat_Ac_iniS) Creatinina (Cr)	
(Ureia_Ac_iniS) Ureia (Ur)	
(TGO_Ac_iniS) Transaminase Glutâmico Oxalacético (TGO)	
(TGP_Ac_iniS) Transaminase Glutâmico Pirúvica (TGP; SGPT)	
(Fosfa_Ac_iniS) Fosfatase Alcalina (ALP)	

8. Dados Fisiológicos:

(FC_Ac_iniS) Frequência Cardíaca (bpm)	
(FR_Ac_iniS) Frequência Respiratória (ipm)	
(PAS_Ac_iniS) Pressão Arterial Sistólica (mmHg)	
(Temp_Ac_iniS) Temperatura (°C)	
(Sat_Ac_iniS) Saturação de O2 (%)	

9. (Ncons_Ac_iniS) Nível de consciência

- ◯ Alerta
- ◯ Confuso
- ◯ Resposta à dor
- ◯ Inconsciente

10. (Rdis_Ac_iniS) Respiração com dispositivo invasivo (TOT, traqueostomia)?

- ◯ Sim
- ◯ Não

III - INÍCIO DO USO DA SONDA E ACOMPANHAMENTO DO PACIENTE

C – VARIÁVEIS TERAPÊUTICAS

Dados relacionados aos medicamentos prescritos <u>24 HORAS APÓS O INÍCIO DO USO DA SONDA</u> ou após a <u>ADMISSÃO</u> (se o paciente for admitido com sonda)

11. (D_24h_aAd)Data:

Data

Data
DD/MM/AAAA

12. (24h_aAd)Medicamento 1

Nome

Apresentação

Dose

Aprazamento

13. (24h_aAd)Dados Medicamento 1

	Forma farmacêutica	Via de administração	Frequência
(24h_aAd)Medicamento 1			

14. (24h_aAd)Medicamento 2

Nome

Apresentação

Dose

Aprazamento

15. (24h_aAd)Dados Medicamento 2

	Forma farmacêutica	Via de administração	Frequência
(24h_aAd)Medicamento 2			

16. (24h_aAd)Medicamento 3

Nome

Apresentação

Dose

Aprazamento

17. (24h_aAd)Dados Medicamento 3

	Forma farmacêutica	Via de administração	Frequência
(24h_aAd)Medicamento 3			

18. (24h_aAd)Medicamento 4

Nome

Apresentação

Dose

Aprazamento

19. (24h_aAd)Dados Medicamento 4

	Forma farmacêutica	Via de administração	Frequência
(24h_aAd)Medicamento 4			

20. (24h_aAd)Medicamento 5

Nome

Apresentação

Dose

Aprazamento

21. (24h_aAd)Dados Medicamento 5

	Forma farmacêutica	Via de administração	Frequência
(24h_aAd)Medicamento 5			

22. (24h_aAd)Medicamento 6

Nome

Apresentação

Dose

Aprazamento

23. (24h_aAd)Dados Medicamento 6

	Forma farmacêutica	Via de administração	Frequência
(24h_aAd)Medicamento 6			

24. (24h_aAd)Medicamento 7

Nome	
Apresentação	
Dose	
Aprazamento	

25. (24h_aAd)Dados Medicamento 7

	Forma farmacêutica	Via de administração	Frequência
(24h_aAd)Medicamento 7			

26. (24h_aAd)Medicamento 8

Nome	
Apresentação	
Dose	
Aprazamento	

27. (24h_aAd)Dados Medicamento 8

	Forma farmacêutica	Via de administração	Frequência
(24h_aAd)Medicamento 8			

28. (24h_aAd)Medicamento 9

Nome	
Apresentação	
Dose	
Aprazamento	

29. (24h_aAd)Dados Medicamento 9

	Forma farmacêutica	Via de administração	Frequência
(24h_aAd)Medicamento 9			

30. (24h_aAd)Medicamento 10

Nome

Apresentação

Dose

Aprazamento

31. (24h_aAd)Dados Medicamento 10

	Forma farmacêutica	Via de administração	Frequência
(24h_aAd)Medicamento 10			

32. (24h_aAd)Medicamento 11

Nome

Apresentação

Dose

Aprazamento

33. (24h_aAd)Dados Medicamento 11

	Forma farmacêutica	Via de administração	Frequência
(24h_aAd)Medicamento 11			

34. (24h_aAd)Medicamento 12

Nome

Apresentação

Dose

Aprazamento

35. (24h_aAd)Dados Medicamento 12

	Forma farmacêutica	Via de administração	Frequência
(24h_aAd)Medicamento 12			

36. (24h_aAd)Medicamento 13

Nome

Apresentação

Dose

Aprazamento

37. (24h_aAd)Dados Medicamento 13

	Forma farmacêutica	Via de administração	Frequência
(24h_aAd)Medicamento 13			

38. (24h_aAd)Medicamento 14

Nome	
Apresentação	
Dose	
Aprazamento	

39. (24h_aAd)Dados Medicamento 14

	Forma farmacêutica	Via de administração	Frequência
(24h_aAd)Medicamento 14			

40. (24h_aAd)Medicamento 15

Nome	
Apresentação	
Dose	
Aprazamento	

41. (24h_aAd)Dados Medicamento 15

	Forma farmacêutica	Via de administração	Frequência
(24h_aAd)Medicamento 15			

42. (24h_aAd)Medicamento 16

Nome	
Apresentação	
Dose	
Aprazamento	

43. (24h_aAd)Dados Medicamento 16

	Forma farmacêutica	Via de administração	Frequência
(24h_aAd)Medicamento 16			

44. (24h_aAd)Medicamento 17

Nome

Apresentação

Dose

Aprazamento

45. (24h_aAd)Dados Medicamento 17

	Forma farmacêutica	Via de administração	Frequência
(24h_aAd)Medicamento 17			

46. (24h_aAd)Medicamento 18

Nome

Apresentação

Dose

Aprazamento

47. (24h_aAd)Dados Medicamento 18

	Forma farmacêutica	Via de administração	Frequência
(24h_aAd)Medicamento 18			

48. (24h_aAd)Medicamento 19

Nome

Apresentação

Dose

Aprazamento

49. (24h_aAd)Dados Medicamento 19

	Forma farmacêutica	Via de administração	Frequência
(24h_aAd)Medicamento 19			

50. (24h_aAd)Medicamento 20

Nome

Apresentação

Dose

Aprazamento

51. (24h_aAd)Dados Medicamento 20

	Forma farmacêutica	Via de administração	Frequência
(24h_aAd)Medicamento 20			

52. (24h_aAd)Medicamento 21

Nome

Apresentação

Dose

Aprazamento

53. (24h_aAd)Dados Medicamento 21

	Forma farmacêutica	Via de administração	Frequência
(24h_aAd)Medicamento 21			

54. (24h_aAd)Medicamento 22

Nome

Apresentação

Dose

Aprazamento

55. (24h_aAd)Dados Medicamento 22

	Forma farmacêutica	Via de administração	Frequência
(24h_aAd)Medicamento 22			

56. (24h_aAd)Medicamento 23

Nome

Apresentação

Dose

Aprazamento

57. (24h_aAd)Dados Medicamento 23

	Forma farmacêutica	Via de administração	Frequência
(24h_aAd)Medicamento 23			

58. (24h_aAd)Medicamento 24

Nome

Apresentação

Dose

Aprazamento

59. (24h_aAd)Dados Medicamento 24

	Forma farmacêutica	Via de administração	Frequência
(24h_aAd)Medicamento 24			

60. (24h_aAd)Medicamento 25

Nome

Apresentação

Dose

Aprazamento

61. (24h_aAd)Dados Medicamento 25

	Forma farmacêutica	Via de administração	Frequência
(24h_aAd)Medicamento 25			

III - INÍCIO DO USO DA SONDA E ACOMPANHAMENTO DO PACIENTE

C – VARIÁVEIS TERAPÊUTICAS

Dados relacionados aos medicamentos prescritos 120 HORAS APÓS O INÍCIO DO USO DA SONDA ou após a ADMISSÃO (se o paciente for admitido com sonda)

62. (D_120h_aAd)Data:

Data

Data

DD/MM/AAAA

63. (120h_aAd)Medicamento 1

Nome

Apresentação

Dose

Aprazamento

64. (120h_aAd)Dados Medicamento 1

	Forma farmacêutica	Via de administração	Frequência
(120h_aAd)Medicamento 1			

65. (120h_aAd)Medicamento 2

Nome

Apresentação

Dose

Aprazamento

66. (120h_aAd)Dados Medicamento 2

	Forma farmacêutica	Via de administração	Frequência
(120h_aAd)Medicamento 2			

67. (120h_aAd)Medicamento 3

Nome	
Apresentação	
Dose	
Aprazamento	

68. (120h_aAd)Dados Medicamento 3

	Forma farmacêutica	Via de administração	Frequência
(120h_aAd)Medicamento 3			

69. (120h_aAd)Medicamento 4

Nome	
Apresentação	
Dose	
Aprazamento	

70. (120h_aAd)Dados Medicamento 4

	Forma farmacêutica	Via de administração	Frequência
(120h_aAd)Medicamento 4			

71. (120h_aAd)Medicamento 5

Nome	
Apresentação	
Dose	
Aprazamento	

72. (120h_aAd)Dados Medicamento 5

	Forma farmacêutica	Via de administração	Frequência
(120h_aAd)Medicamento 5			

73. (120h_aAd)Medicamento 6

Nome	
Apresentação	
Dose	
Aprazamento	

74. (120h_aAd)Dados Medicamento 6

	Forma farmacêutica	Via de administração	Frequência
(120h_aAd)Medicamento 6			

75. (120h_aAd)Medicamento 7

Nome

Apresentação

Dose

Aprazamento

76. (120h_aAd)Dados Medicamento 7

	Forma farmacêutica	Via de administração	Frequência
(120h_aAd)Medicamento 7			

77. (120h_aAd)Medicamento 8

Nome

Apresentação

Dose

Aprazamento

78. (120h_aAd)Dados Medicamento 8

	Forma farmacêutica	Via de administração	Frequência
(120h_aAd)Medicamento 8			

79. (120h_aAd)Medicamento 9

Nome

Apresentação

Dose

Aprazamento

80. (120h_aAd)Dados Medicamento 9

	Forma farmacêutica	Via de administração	Frequência
(120h_aAd)Medicamento 9			

81. (120h_aAd)Medicamento 10

Nome	
Apresentação	
Dose	
Aprazamento	

82. (120h_aAd)Dados Medicamento 10

	Forma farmacêutica	Via de administração	Frequência
(120h_aAd)Medicamento 10			

83. (120h_aAd)Medicamento 11

Nome	
Apresentação	
Dose	
Aprazamento	

84. (120h_aAd)Dados Medicamento 11

	Forma farmacêutica	Via de administração	Frequência
(120h_aAd)Medicamento 11			

85. (120h_aAd)Medicamento 12

Nome	
Apresentação	
Dose	
Aprazamento	

86. (120h_aAd)Dados Medicamento 12

	Forma farmacêutica	Via de administração	Frequência
(120h_aAd)Medicamento 12			

87. (120h_aAd)Medicamento 13

Nome	
Apresentação	
Dose	
Aprazamento	

88. (120h_aAd)Dados Medicamento 13

	Forma farmacêutica	Via de administração	Frequência
(120h_aAd)Medicamento 13			

89. (120h_aAd)Medicamento 14

Nome

Apresentação

Dose

Aprazamento

90. (120h_aAd)Dados Medicamento 14

	Forma farmacêutica	Via de administração	Frequência
(120h_aAd)Medicamento 14			

91. (120h_aAd)Medicamento 15

Nome

Apresentação

Dose

Aprazamento

92. (120h_aAd)Dados Medicamento 15

	Forma farmacêutica	Via de administração	Frequência
(120h_aAd)Medicamento 15			

93. (120h_aAd)Medicamento 16

Nome

Apresentação

Dose

Aprazamento

94. (120h_aAd)Dados Medicamento 16

	Forma farmacêutica	Via de administração	Frequência
(120h_aAd)Medicamento 16			

95. (120h_aAd)Medicamento 17

Nome	
Apresentação	
Dose	
Aprazamento	

96. (120h_aAd)Dados Medicamento 17

	Forma farmacêutica	Via de administração	Frequência
(120h_aAd)Medicamento 17			

97. (120h_aAd)Medicamento 18

Nome	
Apresentação	
Dose	
Aprazamento	

98. (120h_aAd)Dados Medicamento 18

	Forma farmacêutica	Via de administração	Frequência
(120h_aAd)Medicamento 18			

99. (120h_aAd)Medicamento 19

Nome	
Apresentação	
Dose	
Aprazamento	

100. (120h_aAd)Dados Medicamento 19

	Forma farmacêutica	Via de administração	Frequência
(120h_aAd)Medicamento 19			

101. (120h_aAd)Medicamento 20

Nome	
Apresentação	
Dose	
Aprazamento	

102. (120h_aAd)Dados Medicamento 20

	Forma farmacêutica	Via de administração	Frequência
(120h_aAd)Medicamento 20			

103. (120h_aAd)Medicamento 21

Nome

Apresentação

Dose

Aprazamento

104. (120h_aAd)Dados Medicamento 21

	Forma farmacêutica	Via de administração	Frequência
(120h_aAd)Medicamento 21			

105. (120h_aAd)Medicamento 22

Nome

Apresentação

Dose

Aprazamento

106. (120h_aAd)Dados Medicamento 22

	Forma farmacêutica	Via de administração	Frequência
(120h_aAd)Medicamento 22			

107. (120h_aAd)Medicamento 23

Nome

Apresentação

Dose

Aprazamento

108. (120h_aAd)Dados Medicamento 23

	Forma farmacêutica	Via de administração	Frequência
(120h_aAd)Medicamento 23			

109. (120h_aAd)Medicamento 24

Nome	
Apresentação	
Dose	
Aprazamento	

110. (120h_aAd)Dados Medicamento 24

	Forma farmacêutica	Via de administração	Frequência
(120h_aAd)Medicamento 24			

111. (120h_aAd)Medicamento 25

Nome	
Apresentação	
Dose	
Aprazamento	

112. (120h_aAd)Dados Medicamento 25

	Forma farmacêutica	Via de administração	Frequência
(120h_aAd)Medicamento 25			

III - INÍCIO DO USO DA SONDA E ACOMPANHAMENTO DO PACIENTE

C – VARIÁVEIS TERAPÊUTICAS

Dados relacionados aos medicamentos prescritos <u>24h ANTES DA ALTA (ÓBITO/TRANSFERÊNCIA)</u> ou <u>retirada programada da sonda</u>:

113. (24h_aAlt)Data:

Data

Data

DD/MM/AAAA

114. (24h_aAlt)Medicamento 1

Nome

Apresentação

Dose

Aprazamento

115. (24h_aAlt)Dados Medicamento 1

	Forma farmacêutica	Via de administração	Frequência
24h_aAlt)Medicamento 1			

116. (24h_aAlt)Medicamento 2

Nome

Apresentação

Dose

Aprazamento

117. (24h_aAlt)Dados Medicamento 2

	Forma farmacêutica	Via de administração	Frequência
(24h_aAlt)Medicamento 2			

118. (24h_aAlt)Medicamento 3

Nome

Apresentação

Dose

Aprazamento

119. (24h_aAlt)Dados Medicamento 3

	Forma farmacêutica	Via de administração	Frequência
(24h_aAlt)Medicamento 3			

120. (24h_aAlt)Medicamento 4

Nome

Apresentação

Dose

Aprazamento

121. (24h_aAlt)Dados Medicamento 4

	Forma farmacêutica	Via de administração	Frequência
(24h_aAlt)Medicamento 4			

122. (24h_aAlt)Medicamento 5

Nome

Apresentação

Dose

Aprazamento

123. (24h_aAlt)Dados Medicamento 5

	Forma farmacêutica	Via de administração	Frequência
(24h_aAlt)Medicamento 5			

124. (24h_aAlt)Medicamento 6

Nome

Apresentação

Dose

Aprazamento

125. (24h_aAlt)Dados Medicamento 6

	Forma farmacêutica	Via de administração	Frequência
(24h_aAlt)Medicamento 6			

126. (24h_aAlt)Medicamento 7

Nome

Apresentação

Dose

Aprazamento

127. (24h_aAlt)Dados Medicamento 7

	Forma farmacêutica	Via de administração	Frequência
(24h_aAlt)Medicamento 7			

128. (24h_aAlt)Medicamento 8

Nome

Apresentação

Dose

Aprazamento

129. (24h_aAlt)Dados Medicamento 8

	Forma farmacêutica	Via de administração	Frequência
(24h_aAlt)Medicamento 8			

130. (24h_aAlt)Medicamento 9

Nome

Apresentação

Dose

Aprazamento

131. (24h_aAlt)Dados Medicamento 9

	Forma farmacêutica	Via de administração	Frequência
(24h_aAlt)Medicamento 9			

132. (24h_aAlt)Medicamento 10

Nome

Apresentação

Dose

Aprazamento

133. (24h_aAlt)Dados Medicamento 10

	Forma farmacêutica	Via de administração	Frequência
(24h_aAlt)Medicamento 10			

134. (24h_aAlt)Medicamento 11

Nome

Apresentação

Dose

Aprazamento

135. (24h_aAlt)Dados Medicamento 11

	Forma farmacêutica	Via de administração	Frequência
(24h_aAlt)Medicamento 11			

136. (24h_aAlt)Medicamento 12

Nome

Apresentação

Dose

Aprazamento

137. (24h_aAlt)Dados Medicamento 12

	Forma farmacêutica	Via de administração	Frequência
(24h_aAlt)Medicamento 12			

138. (24h_aAlt)Medicamento 13

Nome

Apresentação

Dose

Aprazamento

139. (24h_aAlt)Dados Medicamento 13

	Forma farmacêutica	Via de administração	Frequência
(24h_aAlt)Medicamento 13			

140. (24h_aAlt)Medicamento 14

Nome

Apresentação

Dose

Aprazamento

141. (24h_aAlt)Dados Medicamento 14

	Forma farmacêutica	Via de administração	Frequência
(24h_aAlt)Medicamento 14			

142. (24h_aAlt)Medicamento 15

Nome

Apresentação

Dose

Aprazamento

143. (24h_aAlt)Dados Medicamento 15

	Forma farmacêutica	Via de administração	Frequência
(24h_aAlt)Medicamento 15			

144. (24h_aAlt)Medicamento 16

Nome

Apresentação

Dose

Aprazamento

145. (24h_aAlt)Dados Medicamento 16

	Forma farmacêutica	Via de administração	Frequência
(24h_aAlt)Medicamento 16			

146. (24h_aAlt)Medicamento 17

Nome

Apresentação

Dose

Aprazamento

147. (24h_aAlt)Dados Medicamento 17

	Forma farmacêutica	Via de administração	Frequência
(24h_aAlt)Medicamento 17			

148. (24h_aAlt)Medicamento 18

Nome

Apresentação

Dose

Aprazamento

149. (24h_aAlt)Dados Medicamento 18

	Forma farmacêutica	Via de administração	Frequência
(24h_aAlt)Medicamento 18			

150. (24h_aAlt)Medicamento 19

Nome

Apresentação

Dose

Aprazamento

151. (24h_aAlt)Dados Medicamento 19

	Forma farmacêutica	Via de administração	Frequência
(24h_aAlt)Medicamento 19			

152. (24h_aAlt)Medicamento 20

Nome

Apresentação

Dose

Aprazamento

153. (24h_aAlt)Dados Medicamento 20

	Forma farmacêutica	Via de administração	Frequência
(24h_aAlt)Medicamento 20			

154. (24h_aAlt)Medicamento 21

Nome

Apresentação

Dose

Aprazamento

155. (24h_aAlt)Dados Medicamento 21

	Forma farmacêutica	Via de administração	Frequência
(24h_aAlt)Medicamento 21			

156. (24h_aAlt)Medicamento 22

Nome

Apresentação

Dose

Aprazamento

157. (24h_aAlt)Dados Medicamento 22

	Forma farmacêutica	Via de administração	Frequência
(24h_aAlt)Medicamento 22			

158. (24h_aAlt)Medicamento 23

Nome

Apresentação

Dose

Aprazamento

159. (24h_aAlt)Dados Medicamento 23

	Forma farmacêutica	Via de administração	Frequência
(24h_aAlt)Medicamento 23			

160. (24h_aAlt)Medicamento 24

Nome

Apresentação

Dose

Aprazamento

161. (24h_aAlt)Dados Medicamento 24

	Forma farmacêutica	Via de administração	Frequência
(24h_aAlt)Medicamento 24			

162. (24h_aAlt)Medicamento 25

Nome

Apresentação

Dose

Aprazamento

163. (24h_aAlt)Dados Medicamento 25

	Forma farmacêutica	Via de administração	Frequência
(24h_aAlt)Medicamento 25			

III - INÍCIO DO USO DA SONDA E ACOMPANHAMENTO DO PACIENTE

D - VARIÁVEIS FISIOLÓGICAS ANTES ALTA / TRANSFERÊNCIA / ÓBITO

12h antes alta / transferência / óbito:

164. (D_Fis_12haOT)Data:

Data

Data

DD/MM/AAAA

165. Dados Fisiológicos:

(FC_12aOT) Frequência Cardíaca (bpm)	
(FR_12aOT) Frequência Respiratória (ipm)	
(PAS_12aOT) Pressão Arterial Sistólica (mmHg)	
(Temp_12aOT) Temperatura (°C)	
(Sat_12aOT) Saturação de O2 (%)	

166. (Ncons_12aOT) Nível de consciência:

- Alerta
- Confuso
- Resposta à dor
- Inconsciente

167. (Rdis_12aOT) Respiração com dispositivo invasivo (TOT, traqueostomia)?

- Sim
- Não

D - VARIÁVEIS FISIOLÓGICAS ANTES ALTA / TRANSFERÊNCIA / ÓBITO

24h antes alta / transferência / óbito:

168. (D_Fis_24haOT)Data:

Data

Data

DD/MM/AAAA

169. Dados Fisiológicos:

(FC_24aOT) Frequência Cardíaca (bpm)	
(FR_24aOT) Frequência Respiratória (ipm)	
(PAS_24aOT) Pressão Arterial Sistólica (mmHg)	
(Temp_24aOT) Temperatura (°C)	
(Sat_24aOT) Saturação de O2 (%)	

170. (Ncons_24aOT) Nível de consciência:

- Alerta
- Confuso
- Resposta à dor
- Inconsciente

171. (Rdis_24aOT) Respiração com dispositivo invasivo (TOT, traqueostomia)?

- Sim
- Não

III - INÍCIO DO USO DA SONDA E ACOMPANHAMENTO DO PACIENTE

D - VARIÁVEIS FISIOLÓGICAS ANTES ALTA / TRANSFERÊNCIA / ÓBITO

72h antes alta / transferência / óbito:

172. (D_Fis_72haOT)Data:

Data

Data

DD/MM/AAAA

173. Dados Fisiológicos:

(FC_72aOT) Frequência Cardíaca (bpm)	
(FR_72aOT) Frequência Respiratória (ipm)	
(PAS_72aOT) Pressão Arterial Sistólica (mmHg)	
(Temp_72aOT) Temperatura (°C)	
(Sat_72aOT) Saturação de O2 (%)	

174. (Ncons_72aOT) Nível de consciência:

- Alerta
- Confuso
- Resposta à dor
- Inconsciente

175. (Rdis_72aOT) Respiração com dispositivo invasivo (TOT, traqueostomia)?

- Sim
- Não

III - INÍCIO DO USO DA SONDA E ACOMPANHAMENTO DO PACIENTE

E - DADOS DA RETIRADA PROGRAMADA DA SONDA ENTERAL

176. Data da retirada programada da sonda:

Data

Data

DD/MM/AAAA

177. Principal motivo:

- ◯ Dieta por via oral
- ◯ Alta hospitalar / transferência / óbito
- ◯ Recusa do paciente em manter sonda enteral
- ◯ Gastrostomia / jejunostomia / ileostomia
- ◯ Não se aplica
- ◯ Outro (especifique)

III - INÍCIO DO USO DA SONDA E ACOMPANHAMENTO DO PACIENTE

F – DADOS DA ALTA

178. Data e horário da alta:

Data / Hora

Data: DD/MM/AAAA | Hora: hh mm | AM/PM: -

179. Paciente em uso de sonda enteral no momento da alta?

- ◯ Sim
- ◯ Não

180. Motivo da alta:

- ◯ Domicílio
- ◯ Óbito
- ◯ CTI / UTI
- ◯ Centro Cirúrgico
- ◯ Clínica cirúrgica
- ◯ Outro (especifique)

III - INÍCIO DO USO DA SONDA E ACOMPANHAMENTO DO PACIENTE

181. Observações

