# Supplementary material for: Factors associated with mechanical device-related complications in tube fed patients: A multicenter prospective cohort study
Source: PLoS One. 2020 Nov 19;15(11):e0241849. doi: 10.1371/journal.pone.0241849 (PMC7676660; doi:10.1371/journal.pone.0241849)
Supplement: Attachment — Submitted filename: Response to Reviewers.doc [file pone.0241849.s005.doc]

Ribeirão Preto, 05 October 2020

Dear Editor

**PlosOne**

**EDITORIAL REQUESTS:**

**Comment:** Please ensure that your manuscript meets PLOS ONE's style requirements, including those for file naming.

**Answer:** Thank you for your comment. We revised carefully the entire manuscript to guarantee that it meets PlosOne’s style requirements, including those for file naming. In addition, abstract was reduced to keep up to 300 words.

**Comment:** Please include additional information regarding the data collection instruments used in the study and ensure that you have provided sufficient details that others could replicate the analyses.

**Answer:** Thank you for your comment. We included additional information regarding data collection forms (See page 6-7) and a copy of each as Supporting Information (S1-S3 Files).

**Comment:** For instance, if you developed a questionnaire as part of this study and it is not under a copyright more restrictive than CC-BY, please include a copy, in both the original language and English, as Supporting Information.

**Answer:** Thank you for your comment. We provided a copy of each data collection form (S1-S3 Files) in PDF because they are electronic and were developed using the Survey Monkey platform. As described in the methods section, the instruments were submitted to face and content validation process by a panel of experts (See page 7), so they can only be used in the original language. For this reason, it is not possible to provide a copy in English.

**Comment:** In your Methods section, please provide additional information about the participant recruitment method and the demographic details of your participants.

**Answer:** Thank you for your suggestions. We provided additional information about the participant recruitment method and the demographic details of participants (See pages 5-6).

**Comment:** Please ensure you have provided sufficient details to replicate the analyses such as:

a) the recruitment date range (month and year),

b) a table of relevant demographic details and

c) a statement as to whether your sample can be considered representative of a larger population.

**Answer:** Thank you for your comments. The recruitment date range (month and year) information was provided in the data collection section (See page 7). We also included a paragraph with relevant demographic details (See pages 5-6) and the dataset was provided as supporting information (S1 Dataset). A statement as to whether the sample can be considered representative of a larger population was included (See page 5).

**REVIEWER 1**

**Comment:** The authors assessed the types of nasogastric/nasoenteric feeding tube (NGT/NET)-related adverse events, the degree of harm, and to analyze the factors associated with mechanical events. The strength of this study is that it is a prospective and multi-center research. However, the disadvantage of this study is that it lacks novelty.
Additionally, the authors should consider the differences between facilities.

**Answer:** Thank you for your comments. We reinforce the novelty of this study in the discussion session (See page 17). Previous studies showed that patients using NGT/NET are subject to AEs in the hospital setting, but none aimed to assess the degree of harm. This is the first study to do so with this patient profile. In relation to the differences between facilities, we included a sentence considering it as a study limitation (See page 22).

**REVIEWER 2**

**Comment:** Overall, the research is well described, and the supporting information is enough to elucidate about the frequency and risk factors of mechanical adverse effects cause by NGT/NET placement and use. Taking into the account the frequency of NGT/NET use, the number of studies on the topic is surprising low, therefore research on the topic is welcomed. The manuscript is suitable for publication in this journal subject to major revisions.

**Answer:** Thank you for your comments.

**MAJOR REVISIONS**

**Comment:** The manuscript is essentially about the occurrence and factors associated with mechanical AEs (96%) related to NGT/NET. The multivariate analysis was done to identify predictors of mechanical AEs. The entire manuscript, including the title must reflect this. For instance, the title must be changed to “Factors associated with nasogastric/nasoenteric feeding tube related mechanic complications: a multicenter prospective cohort study”. A brief description of other non-mechanical AEs (results) is sufficient.

**Answer:** Thank you for your comments and suggestion. We revised carefully the entire manuscript and the terms used in the entire text. Based on your comment and on our revision, we changed the title of the manuscript. In addition, we included a brief description about the types of NGT-NET-related AEs in the methods section (See page 8).

**Comment:** Page 5, line 118 - Study design – the authors must include information on the inclusion process of the patients (consecutive ? …).

**Answer:** Thank you for your comment. We included a sentence about patient enrollment in the methods section (See page 6).

**Comment:** Page 9, 213 – “Comparisons between the means of the variables “number of comorbidities” at the time of AE and “degree of harm” were performed using the Jonckheere-Terpstra test [20,21]” - The number of comorbidities is a gross measurement of comorbidities burden. The authors should explain the option of not using the CCI which is a much sensitive instrument.

**Answer:** Thank you for your comment. We included a new table (Table 4) with comparisons between the degree of harm and the following variables: care complexity (according to PCS), severity (according to the CCI) and level of consciousness (according to the ACDU scale), instead of comparing the means of the variables “number of comorbidities” at the time of AE and “degree of harm (See pages 14-15).

**Comment:** Page 3, 64 - “can be avoided through good practices in nursing – this conclusion is not supported by the study data. The authors should be less pragmatic.

**Answer:** Thank you for your comment. The sentence was excluded from the abstract (See page 3).

**MINOR REVISIONS**

**Comment:** The manuscript requires significant proof reading and revision to improve the quality of English.

**Answer:** Thank you for your comment. Major revision was conducted in the entire manuscript to improve it. In addition, the document was revised by a native English speaking person to improve the quality of English.

**Comment:** Page 4, line 88 - define light treatment.

**Answer:** Thank you for your suggestion. The entire paragraph was revised and rewritten according to the WHO’s International Classification for Patient Safety (See page 8).

**Comment:** Page 4, line 91 - explain how life expectancy was evaluated in relation to the AEs.

**Answer:** Thank you for your suggestion. The degree of harm was determined according to the WHO international classification for patient safety. The entire paragraph was revised and rewritten to make it clearer (See page 8). In addition, The degree of harm was assessed based on the information contained in the patient’s medical notes and the information obtained from the patient and/or caregiver, nursing and medical teams and medical records (medical and nursing records/developments). It is described in the methods section (See page 8).

**Comment:** Page 5, line 125 – “with and without residency programs” – this is not a necessary information.

**Answer:** Thank you for your comment. The information was excluded from the sentence (See page 4).

**Comment:** The main adverse effect was unplanned/accidental withdrawal (70.1%) – which seems to be the a major AE in non -cooperative patients, for example in stroke patients (Complications Associated With Nasogastric Tube Placement in the Acute Phase of Stroke: A Systematic Review.Nascimento A, Carvalho M, Nogueira J, Abreu P, Nzwalo H.J Neurosci Nurs. 2018 Aug;50(4):193-198. doi: 10.1097/JNN.0000000000000372.). Discussion of the implications of NGT in non-cooperative patients should be included.

**Answer:** Thank you for your suggestion. A paragraph with the implications of NGT in non-cooperative patients was included (See page 20).
